# Adaptive neurons compute confidence in a decision network

**DOI:** 10.1038/s41598-021-01523-9

**Published:** 2021-11-12

**Authors:** Luozheng Li, DaHui Wang

**Affiliations:** 1 School of Systems Science and State Key Lab of Cognitive Neuroscience and Learning, Beijing Normal University, Beijing, 100875 China; 2 Beijing Key Laboratory of Brain Imaging and Connectomics, Beijing Normal University, Beijing, 100875 China; 3 Wangxuan Institute of Computer Technology, Peking University, Beijing, 100080 China

**Keywords:** Decision, Computational neuroscience, Network models

## Abstract

Humans and many animals have the ability to assess the confidence of their decisions. However, little is known about the underlying neural substrate and mechanism. In this study we propose a computational model consisting of a group of ’confidence neurons’ with adaptation, which are able to assess the confidence of decisions by detecting the slope of ramping activities of decision neurons. The simulated activities of ’confidence neurons’ in our simple model capture the typical features of confidence observed in humans and animals experiments. Our results indicate that confidence could be online formed along with the decision formation, and the adaptation properties could be used to monitor the formation of confidence during the decision making.

## Introduction

In our daily lives, we often estimate the confidence of our perceptions and decisions. Confidence, a kind of metacognitive process, not only reflects the subjective assessment of our choice^1,2^, but also implies monitoring of our own cognitive process^3–5^. Neural correlates to the confidence have been revealed by many experiments, for examples, neurons in parietal cortex of monkey represented formation of the decisions and the confidence of the decisions^6^; single neuron in the human medial temporal lobe represented the retrieval confidence^7^ and the activities of single neuron in the same area were persistently correlated with decision confidence^8^; some neurons in the orbitofrontal cortex of rats positively tuned confidence encoding^9^; the functional magnetic resonance imaging signal in the human ventromedial prefrontal cortex reflected both value comparison and confidence in the value comparison process^10^; an area in the medial prefrontal cortex called the perigenual anterior cingulate cortex signaled confidence^11^. Besides the positive correlations between the neural activities and confidence, the neural activity can be negatively correlated with confidence. For examples, the activation of the right dorsolateral prefrontal cortex in humans was greater for low-confidence than that for high-confidence^12^; the firing rates of some single neuron or population activities in the orbitofrontal cortex of rats are positively correlated with uncertainty^2,5^, where uncertainty can be mathematically thought of as the opposite of confidence, i.e. the larger uncertainty implies lower confidence, and vice versa. Although many neural correlates have been found, it is still unknown how the confidence forms on the neural circuit level during the decision process.

Theoretical models also have also tried to explore the computation of confidence in the brain. One type of models think of neural responses as the probability distributions and of confidence as quantifiable by evaluating the posterior probability^13,14^. These models capture statistical characteristics of decision confidence but lack neurobiological interpretability. Another type of models define the confidence as the absolute difference between the firing rates of neuron population selective to the decision options at decision time, where the firing rates are produced either by race model^10^ or dynamic attractor model^15^. The race model based confidence explains the activation of the human ventromedial prefrontal cortex^10^ and the dynamic attractor model based confidence successfully reproduce the observations in monkey experiments^6^ and human confidence in a sequence of perceptual decisions^16^ . However, how the neural circuit calculates the absolute difference between neuron pools, i.e., how the confidence forms during the decision, is unclear. The third type of models assume that decisions are made by many loosely coupled modules, each of which represents a stochastic sample of the sensory evidence integral, and the confidence is encoded in the dispersion between modules^17^. But, these models do not explain how neural system reads out the dispersion between modules. The fourth type of models use one population of neurons to monitor the activities of decision neurons and produce the uncertainty signal^18–20^. While this type of models successfully explains the electrophysiological recording data from the orbitalfrontal cortex of rats^2^ and the phenomena of change-of mind^21^, these models did not directly explain the formation of confidence since uncertainty can be thought of as the opposite of confidence.

In the present study, we attempt to directly explain the formation of confidence during the decision process based on ann attractor model of decision making. The model consists of a classical decision module^22,23^ and a confidence module. The confidence module receives the inputs from decision neurons. The activities of the confidence module can represent the confidence observed in experiments. In order for the confidence module to calculate the confidence, we introduced the spike frequency adaptation to the confidence neurons. Mathematically, the adaptation enables the neurons to detect the slope of ramping activities of decision circuits^24^. Thus, confidence computation and decision making can be implemented in one simple neural circuit.

## Results

### Model structure

**Figure 1:**
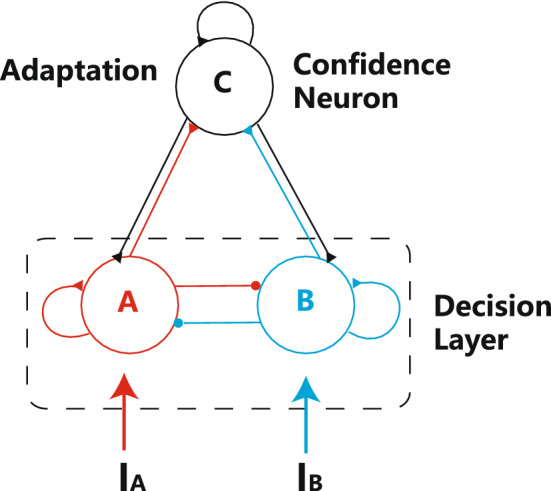
Model structure. The model consists of a decision layer and a confidence neuron pool. The decision layer follows the classical decision circuit^22,23,27^. The adaptive confidence neurons receive the feedforward input from two competing neuron pools in the decision layer and send the feedback projections to the decision layer.

The model consists of two modules: a classical decision circuit and a confidence module which includes recurrent connected neurons(as shown in Fig. 1). The decision module has been well discussed in previous studies on two-alternative choice tasks^25–27^. It consists of two groups of competing neurons (A and B), and both groups receive feedforward inputs from upstream neurons and feedback currents from the confidence neurons. The confidence module (C) consists of one group of neurons whose activities reflect the confidence of decisions. Neurons in the confidence module are innervated by both neural groups in the decision circuit (A and B) and send feedback projections to the decision module. The confidence evaluates the decisions process regardless of the winner among the options and each population of neurons in the decision module has the same influence on the confidence module. Thus, each decision neuron projects to the confidence neuron with the same synaptic conductance.

### Ramping activities in the decision module

**Figure 2:**
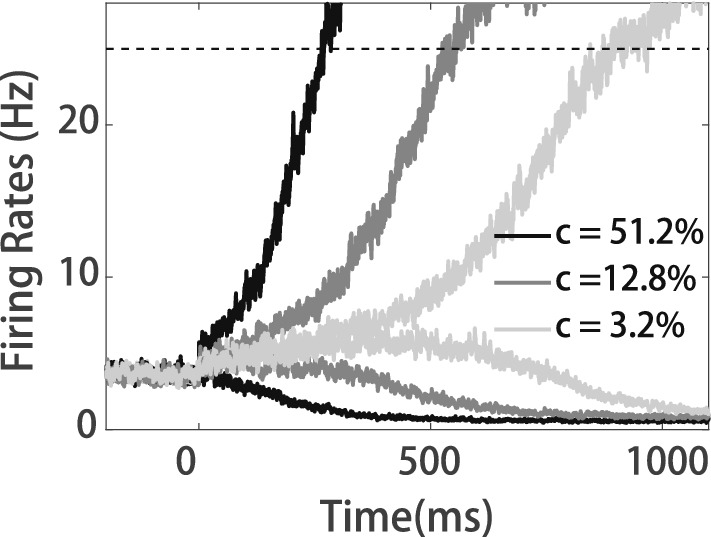
Ramping activities of the neurons in the decision layer during decision making. The dashed line indicates the decision threshold.

We use our model to simulate a simple random dots motion task as described in previous decision models^22,23^. In the decision module, firing rates of neurons displayed the ramping activity during the stimulus presentation before the decision was made (Fig. 2), which is consistent with previous electrophysiological^28^ and theoretical studies^22,23^. The larger value of *c* stands for the stronger evidence or the easier task, leading to steeper ramping activity (as shown in Fig. 2) and shorter decision time. At the same time, the larger *c* and shorter decision time imply an easier task where the subject should show higher confidence in the experiment. Thus, the slope of ramping activities can be thought of as a signal of confidence in the decision^1^. If the downstream neural circuit can detect the slope of ramping activities, the confidence signal can be measured.

### Activities of confidence neurons

In our model, the confidence neurons are designed to detect the slope of ramping activities of decision neurons through an adaptation mechanism. The neurons receive excitatory feedforward inputs from the decision layer, as well as inhibitory currents caused by spike frequency adaptation. Based on biological evidence, adaptive currents will increase with the firing rates of confidence neurons with a time delay^29^ (see Eq. 9). Thus, the activities of confidence neurons first increase along with the ramping activities of the decision layer and then elicit inhibitions caused by the adaptive current. When the stimulus is easily to be discriminated, ramping activities in decision layer have a large slope (see Fig. 2). Because of adaptive current’s large time constant ($\tau _a$ in Eq. 9), the inhibitory adaptive currents(*a*(*t*)) cannot keep pace with the increasing inputs caused by the decision neurons’ rapid ramping activities ($r_{in}(t)$). As a result, with the integration of time (see Eq. 7), the confidence neurons receive weak inhibitory current caused by adaptation and reach a higher firing rate at the decision moment. In contrast, when a difficult task is given, the inhibitory adaptive current (*a*(*t*)) can catch up with the inputs from the decision neurons’ ramping up activities ($r_{in}(t)$), which leads to a larger inhibitory adaptive current and a lower firing rate at the decision moment.

**Figure 3:**
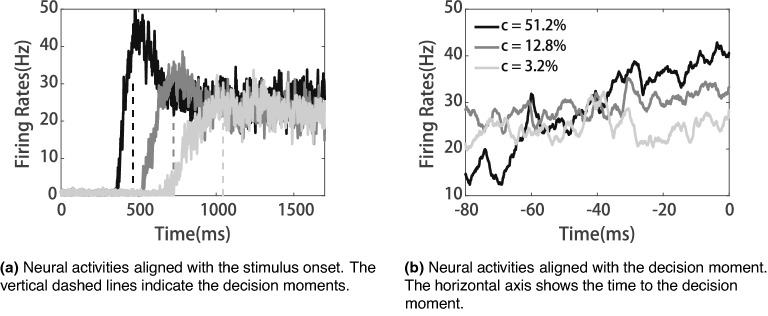
Activities of confidence neurons during decision making.

In the simulations, we defined the decision moment following a previous study’s convention^23^: once the ramping activity of the decision circuit exceeds the decision threshold (25 Hz), the network makes a choice. Figure 3a shows exemplary trials of the activities of confidence neurons. The firing rates of confidence neurons ramp up to different levels based on the tasks’ difficulty levels (*c*). Steeper ramping activities of the decision neuron (larger *c*) correspond to a higher firing rate of the confidence neuron at the decision moment. For clarity, we also aligned the time of confidence neurons’ firing rates to the decision moment (Fig. 3b ). With more precise time scales, we can clearly see that the activities of confidence neurons are negatively correlated with the task difficulty at the decision time (Fig. 3b).

### Typical features of reports by confidence neurons

**Figure 4:**
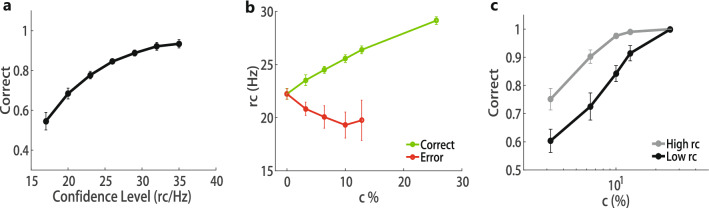
Simulated confidence. The reported confidence (*rc*) by confidence neuron is consistent with the reported confidence in human experiments. Error bars show the standard error for 10 sessions. (**a**). The correct ratio as an increasing function of confidence. (**b**). The confidence increases with the strength of evidence for the trials whose reports are correct but decreases with the strength of evidence for trials whose reports are incorrect. (**c**). The ratio of correct report of the trials with higher confidence is higher than that with lower confidence given the same evidence.

Activities of confidence neurons may be affected by noise in a single trial, so it is necessary to analyze their statistical behaviors. In the simulations, the confidence report or neural representation of confidence, *rc*, is represented by the mean firing rates of confidence neurons in the interval of 10*ms* just before the decision moment. Simulation results reveal that the statistical behaviors of *rc* (Fig. 4) is consistent with the typical features of the general confidence as reported in human and animal experiments^1,5,9,30,31^ . Firstly, the decision accuracy is positively correlated with the confidence level (based on indirect measurement or direct report in experiment) (Fig. 4a). Secondly, by splitting the trials into correct and incorrect trials, it can be found that the confidence level of trials with correct decisions will increase as the task difficulty decreases, and the opposite results is obtained on the error trials (Fig. 4b).Thirdly, the psychometric curve of trials which report high confidence shifts upward (Fig. 4c). In brief, the activities of confidence neurons in our circuit model behave like the general confidence observed in experiments, suggesting that the activities of confidence neurons in our model could represent and compute the confidence in the decision.

**Figure 5:**
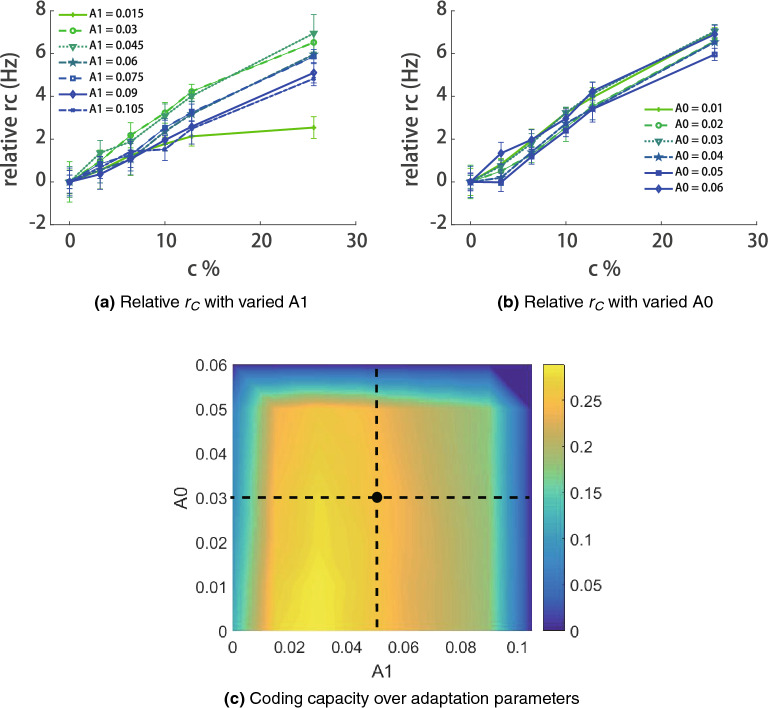
The influence of adaptation parameters on confidence representation. (**a**) The effects of *A*1; (**b**) The effects of *A*0; (**c**) The dependence of coding capacity on adaptation parameters.The error bars in (**a**) and (**b**) show the standard error of 10 sessions. Different colors in (**c**) code the average slope of rc.

### Sensitivity of adaptation on the coding of confidence

Adaptation of confidence neurons plays a key role in the confidence computation during the decision process. In our model, neural adaptation is described by two parameters (in Eq. 9), $A_1$, reflecting the strength of the adaptation caused by spikes, and $A_0$ indicating the baseline level of adaptation in the resting state. Since different adaptation strength causes different firing rates of the confidence neurons at random level ($\%c = 0$), we use ’relative *rc*’ ($rc - rc(\%c = 0 )$) instead of *rc* to denote the changes of slopes. To investigate the influence of adaptation parameters on the confidence coding, we calculate ’relative *rc*’ over the different coherence levels given varied $A_1$ and $A_0$. In Fig. 5a, we plot the curves with different $A_1$ and fixed $A_0$, and similar curves are shown in in Fig. 5b for different $A_0$ and fixed $A_1$.

The curves’ slope in Fig. 5a and b reflect the coding capability of confidence neurons. A slope of zero means the confidence neurons have the same firing rates given stimuli with different coherence levels, which implies that confidence neurons cannot code the decision confidence. Larger slopes indicate larger difference in confidence neurons’ firing rates between stimuli with different coherence levels, which means that confidence neurons are more sensitive to the changes in confidence. Figure 5c shows the dependence of the slope on parameters $A_1$ and $A_0$, where a horizontal dashed line is shown in Fig. 5a and vertical lines are shown in Fig. 5b. These results indicate that the adaptation modulation is statistically robust, because that a large range of parameters values (yellow areas in Fig. 5c) support the confidence coding.

## Discussion

In this study, we propose a computational model in which the decision confidence can be computed and represented in a simple neural circuit. We suggest that the representation of confidence can be achieved by neural adaptation which provides common negative feedbacks in the neural system. Based on the previous observations in experiments^2,5^ and theoretical models of decision making^22,23,27^, we designed the confidence neurons as one neural group whose activities reflect the decisions’ confidence level of the d. Our simulation results confirm that the activities of confidence neurons successfully capture the general features of confidence consistently documented in animals and human behavioral experiments^1,5,30,31^. At last, we investigated the influence of adaptation parameters on the confidence coding, and demonstrated that the adaptation modulation is statistically robust.

For this study, the following points are worth noting. Firstly, we used one specific group of neurons to compute and represent the confidence during the decision making, which is supported by a number of studies. One recent experiment identified that single neuron in the orbitofrontal cortex of rats can encode general decision confidence^9^. Some neurons in the orbitofrontal cortex of rats reflect uncertainty during decision making^2,5^. A single neuron in human medial temporal lobe was found signaling the confidence during decision making^7,8^. Since the confidence was computed by one specific group of neurons, the confidence formation should be thought as a secondary neural processing based on the activities of decision neurons and the decisions process. Thus, the confidence in our model is in our model is a type of second-order cognition in the perspective of the neurophysiology^32^. However, in our model, confidence is formed simultaneously formed along with the decision making, so the post-decision information cannot be considered in a retrospective way^33^ and the empirical dissociations of error detections are not observed in the model.

Secondly, results of our model are consistent with the experimental observations that choice accuracy and confidence reporting are separated processes^5,6,34^,suggesting that confidence computation may not be accomplished in the decision layer. At the same time, our model is different from the notion that neural system may encode the confidence in the form of reaction time^5^. Actually, experiments showed that the reaction time cannot fully account for confidence reports^1^. In our model, the decision confidence was computed in neural circuits without extra decoding strategy, which is simpler but biologically plausible.

Thirdly, confidence is negatively related with uncertainty, i.e., higher uncertainty implies lower confident, and vice versa. The underling neural mechanism of uncertainty was investigated using a computational model consisting of one decision module and one uncertainty monitoring module^18–20^. These models not only explain the formation of uncertainty but also predict the change-of-mind during the decision making^18^ and even after the decision^19^. The key mechanism of the uncertainty model is that the uncertain neuron pool was inhibited by the decision module via a group of inhibitory neurons and received topdown tonic excitation from another cortical area^18,19^. Although uncertainty was mathematically thought of as the opposite of confidence, the two metrics cannot be considered equivalent and the uncertainty cannot be translated into confidence through a simple action mapping. Actually, confidence has its own neural correlates and uncertainty has its own neural correlates, too. For example, single neuron in human medial temporal lobe positively signals the confidence^7,8^, and the perigenual anterior cingulate cortex encodes the confidence, while the activities of some neurons in the orbitofrontal cortex of rats were positively correlated with uncertainty but not with confidence^2,5^. Thus, our brain may have complementary neural substrate to monitor the confidence and uncertainty. Lower confidence and higher uncertainty may elicit a change-of-mind, while higher confidence and lower uncertainty will result in persistence in the current opinion or action. Besides confidence and uncertainty, our brain has other complementary neural circuits to implement the complementary cognitive functions. For examples, unexpected rewards/gains and unexpected punishments/losses are respectively represented by phasic activity of dopaminergic neurons^35^ and the lateral habenular neurons^36^; concurrent multisensory integration and segregation can be implemented by the complementary congruent and opposite neurons^37^. In brief, the confidence and uncertainty may have distinct but interacting neural circuits and the future computational research should combine these two complementary circuits into one model and account for phenomena in confidence and uncertainty.

**Figure 6:**
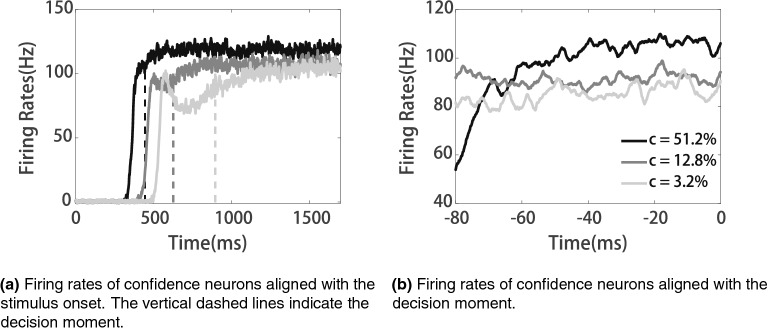
Activities of confidence neurons during decision making given synaptic short-term depression.

Fourthly, in the present model, adaptation is spike frequency dependent, which is an adaptation at the single-neuron level. However, adaptation can happen at the synaptic level, such as short-term depression for instance. Figure 6shows the activities of confidence neurons where the synapses from decision module to confidence module are short-term depressed. The activities of confidence neurons are negatively correlated with task difficulty at the decision time, which is similar to the findings presented in Fig. 3. Thus, different types of adaptation may have similar results.

**Figure 7:**
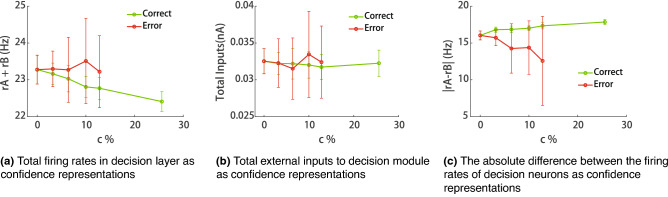
Performance of confidence representation variants.

Fifthly, one may think that confidence could be represented by the summation of firing rates of the decision module ($r_A +r_B$) or by total inputs into the decision module ($I_A +I_B+I_{noise}$), since the confidence neurons in our model receive excitatory inputs from two groups of neurons of decision module. However, we found that total firing rates of the decision module ($r_A +r_B$) and total inputs into the decision module ($I_A +I_B+I_{noise}$) cannot capture the typical feature of confidence reported in human and animal experiments (Fig. 7a,b), suggesting that confidence computation is a non-trivial process. Furthermore, we found that the absolute difference in firing rates of decision neurons $(|r_A-r_B|)$ at the decision time can capture the typical feature of confidence (Fig. 7c) as reported in previous studies^10,15^,but the sensitivity is not as good as that of our model due to the smaller dynamic range of the firing rate of the loser population at the decision moment.

At last, while it is beneficial that our model is simple, it shouldn’t be overly simple. Our model has some limitations The model’s key mechanism is the detection of the slope of decision neurons’ ramping activity. If the ramping activities of decision neurons disappeared or were disturbed by some factors, the proposed mechanism may become invalid. Moreover, confidence was formed simultaneously along with the decision making, additional elements such as uncertainty implicated circuit should be introduced into the model to simulate the complex decision tasks and reveal the underlying mechanism of change-of-mind and the post-decision evaluations of the decisions.

## Methods

### Dynamics of the decision circuit

The spiking neuron model^22^and reduced mean-field model^23^ were proposed in the previous theoretical studies to explain the mechanisms underlying binary decisions. The spiking neuron model is more biological while the mean-filed model is concise and convenient for theoretical analysis. Both types of model successfully replicated the majority of the psychophysical and physiological results in the monkey experiments^23,27^. In this study, we adopt the mean-field model to describe the neural dynamics in the decision circuit. As described in previous work^22,23,27^, the dynamics of neurons in the decision module can be described by the slow dynamics of N-methyl-D-aspartic acid(NMDA) receptors:

1 $$\begin{aligned} \frac{dS_i}{dt}= & {} -\frac{S_i}{\tau _{NMDA}} + (1 - S_i)\gamma r_i , \end{aligned}$$

where $S_i$ is the gating variable of NMDA, *i* is A or B, standing for the group label. $\tau _{NMDA}$ is the decay time constant of NMDA. $\gamma$ is a constant that controls the strength of the gain of $S_i$ caused by firing rates. $r_i$ represents the firing rates of the two neural population. The dynamics of $r_i$ are given by:

2 $$\begin{aligned} r_i= & {} \phi (I_{syn,i}), \end{aligned}$$

3 $$\begin{aligned} I_{syn,i}= & {} J_{ii}S_i - J_{ij}S_j + I_0 + I_{ext,i} + J_{fc}r_C + I_{noise,i}, \end{aligned}$$

where $\phi (x)$ is the input-output function of the single neuron, describing the relation between synaptic input current and neural firing rate. $I_{syn,i}$ represents the synaptic currents of the neural group *i*(A or B). $J_{ii}$ and $J_{ij}$ are the strength of recurrent connections and cross inhibition, respectively. $I_0$ is the background input without bias, while $I_i$ is the stimulus to the population *i* with varied strength. $J_{fc}$ is the synaptic strength of the feedback connections from the confidence neurons(C). $I_{noise,i}$ is a noise term.

As in the previous studies^23^, the function $\phi (x)$ is chosen as:

4 $$\begin{aligned} \phi (I) = \frac{c_EI - I_{th}}{1 - \mathrm{exp}[-g_E(c_EI - I_{th})]}, \end{aligned}$$

where $c_E$ is the gain factor, $I_{th}$ is the threshold current, and $g_E$ is a noise factor determining the nonlinearity of the function.

### Dynamics of confidence neurons

We considered a group of neurons that receive inputs from the decision circuit. The group is named ’confidence neurons’ since we can read out the confidence of decision according to its activities. The dynamics of the confidence neurons are similar to neurons in the decision module, except for the adaptation currents,

5 $$\begin{aligned} \frac{dr_C}{dt}= & {} -\frac{r_C}{\tau _r} + \phi _C( I_{syn,C}), \end{aligned}$$

6 $$\begin{aligned} \frac{dS_C}{dt}= & {} \frac{-S_C}{\tau _{NMDA}} + \gamma (1-S_C)r_C, \end{aligned}$$

7 $$\begin{aligned} I_{syn,C}= & {} J_{C}S_C + J_{dc}r_{in}+ I_{0c} - J_aa + I_{noise,C}, \end{aligned}$$

where $r_C$ is the firing rate of the confidence neuron, and $\tau _r$ the time constant of the firing rate, usually $2-5 ms$. $\phi _C$ describes the input-output function of the confidence neurons, which is simplified as:

8 $$\begin{aligned} \phi _C(I) = \mathrm{max}(c_EI - I_{th,C}, 0.5), \end{aligned}$$

$I_{syn,C}$ is the synaptic currents, and $S_C$ is the gating variable of NMDA. $J_{C}$ denotes the strength of the recurrent connection between confidence neurons. $r_{in} = r_A + r_B$, indicates the inputs from the decision layer. $J_{dc}$ is the connection strength from the decision layer to the confidence neurons. Adaptation currents are denoted by *a* and controlled by the constant $J_a$.

Adaptation is very common in the nervous system. Previous studies revealed that many cellular mechanisms can contribute to the neural adaptation. These mechanisms can be divided into two classes^38,39^: the spike-triggered mechanisms, e.g., the calcium-activated potassium current, and the subthreshold voltage-dependent mechanisms, e.g., the voltage-gated potassium current. Here we model adaptation currents based on these two general mechanisms and the adaptation current of the confidence neuron is given by:

9 $$\begin{aligned} \frac{da}{dt}= & {} -\frac{a}{\tau _a} + A_1r_{C} + A_0, \end{aligned}$$

where $\tau _a$ is the time constant of adaptation and reflects the slow dynamics of calcium currents. Parameter $A_1$ denotes the strength of adaptation caused by spikes, while $A_0$ is the strength of subthreshold adaptation.

### Short-term depression as adaptation

To demonstrate that adaptation is a general mechanism for confidence computation, we used synaptic short-term depression (STD) as an alternative for the spike frequency adaptation. The dynamics of confidence neurons can be rewritten as:

10 $$\begin{aligned} \frac{dr_C}{dt}= & {} -\frac{r_C}{\tau _r} + \phi _C( I_{syn,C}), \end{aligned}$$

11 $$\begin{aligned} \frac{dS_C}{dt}= & {} \frac{-S_C}{\tau _{NMDA}} + \gamma (1-S_C)r_C, \end{aligned}$$

12 $$\begin{aligned} I_{syn,C}= & {} J_{C}S_Cx + J_{dc}r_{in}+ I_{0c} + I_{noise,C}, \end{aligned}$$

where *x* is the normalized depression variable, denoting the fraction of resources that remain available after neurotransmitter depletion. The dynamics of *x* follow previous studies^40,41^:

13 $$\begin{aligned} \frac{dx}{dt}= & {} \frac{(1-x)}{\tau _d} - U_0xr_C, \end{aligned}$$

where $\tau _d$ is the time constant of STD. $U_0$ is a strength constant, standing for the fraction of available resources ready for use.

## Simulation protocol

We simulate the general two-alternative forced choice in a decision-making task with reaction-time style. Many similar experiments were performed with monkeys^28^ and humans^1^.

**Table 1:** Parameters used in the model.

Parameter	Value
$\tau _{NMDA}$, time constant of NMDA receptors	0.1 s
$\tau _a$, time constant of adaptation	0.25 s
$\tau _r$, time constant of firing rate	0.002 s
$\tau _d$, time constant of STD	1 s
$\theta$, decision threshold	25 Hz
$\gamma$, NMDA gain factor per spike	0.641
$J_{ii}$, synaptic strength within neural groups	0.2609 nA
$J_{ij}$, synaptic strength between neural groups	0.0497 nA
$J_C$, synaptic strength between confidence neurons	0.15 nA
$J_{fc}$, feedback synaptic strength from confidence neurons	0.0002 nA
$J_{ext}$, external input synaptic strength to decision layer	0.15 nA
$J_{dc}$, feedforward synaptic strength	0.015 nA/Hz
$J_a$, gain of adaptive currents to confidence neuron	0.001
$c_E$, slope of the F-I function of decision neurons	270/(VnC)
$I_{th}$, firing threshold of decision neurons	108 Hz
$g_E$, noise factor of decision neurons	0.154 s
$I_{th,C}$, threshold of confidence neurons	108 Hz
$A_1$, strength of adaptation caused by spikes,	0.05 nA
$A_0$, strength of subthreshold adaptation	0.03 nA Hz
$\mu _0$, average external inputs	30 Hz
$U_0$, release probabilities in STD	0.0001
$I_0$, background inputs in decision layer	0.3255 nA
$I_{0c}$, background inputs in confidence neurons	0.2 nA

The second order Runge–Kutta method with an time step of 0.05 ms is applied for numerical simulations. Parameters in the simulations are chosen as shown in Table  1 without specification.

In a single trial, we simulate the model for a fixed time period $T = 1500\, ms$. The network receives only unbiased background inputs from $t = -200\, ms$ to $0\, ms$. Biased stimulus is onset at $t = 0\, ms$, and the decision circuit receives external inputs from $t = 0\, ms$ to $t = 1000\, ms$, The stimulus is set as biased inputs as in^23^:

14 $$\begin{aligned} I_{ext} = J_{ext}\mu _0 \left(1 \pm \frac{c}{100\%} \right), \end{aligned}$$

where *c* stands for the task difficulty, which is the coherence level in a dot-motion task^28^, larger *c* value corresponds to an easier trials. $J_{ext}$ is the average synaptic coupling with AMPAR receptors, $\mu _0$ stands for the absolute stimulus strength. Decisions are made when the firing rates of the two competing neural groups reaches a threshold ($\theta = 25 \, Hz$).

To compare with the experimental results, we calculated the average value of $r_C$ across an interval of 10 ms before the decision time as the indicator of confidence. To investigate the statistic features of activities of the confidence neurons, we employ 10 sessions, with 500 trials each. For the simulations of each value of the adaptation parameters, we also employ 10 sessions, with 500 trials each.
